# Detection of Potentially Diagnostic *Leishmania* Antigens with Western Blot Analysis of Sera from Patients with Cutaneous and Visceral Leishmaniases

**Published:** 2017

**Authors:** Seyyed Javad SEYYEDTABAEI, Ali ROSTAMI, Ali HAGHIGHI, Mehdi MOHEBALI, Bahram KAZEMI, Shirzad FALLAHI, Adel SPOTIN

**Affiliations:** 1. Dept. of Parasitology and Mycology, School of Medicine, Shahid Beheshti University of Medical Sciences, Tehran, Iran; 2. Student Research Committee, Shahid Beheshti University of Medical Sciences, Tehran, Iran; 3. Dept. of Medical Parasitology & Mycology, School of Public Health, Tehran University of Medical Sciences, Tehran, Iran; 4. Center for Research of Endemic Parasites of Iran (CREPI), Tehran University of Medical Sciences, Tehran, Iran; 5. Dept. of Biotechnology, Shahid Beheshti University of Medical Sciences, Tehran, Iran; 6. Dept. of Parasitology and Mycology, School of Medicine, Lorestan University of Medical Sciences, Khorramabad, Iran; 7. Dept. of Parasitology and Mycology, School of Medicine, Tabriz University of Medical Sciences, Tabriz, Iran

**Keywords:** Visceral leishmaniasis, Cutaneous leishmaniasis, Diagnostic antigens, Western blot, IFAT

## Abstract

**Background:**

Visceral leishmaniasis (VL) and cutaneous leishmaniasis (CL) are important public health problems in Iran. We aimed to evaluate the diagnostic potential of Western blot (WB) compared with indirect immunofluorescence test (IFAT) to serodiagnosis of leishmaniasis.

**Methods:**

This study was performed from 2010–2014 and participants were different parts of Iran. Serum samples were obtained from 43 patients with proven CL, 33 patients with proven VL, 39 patients with other parasitic diseases and 23 healthy individuals.

**Results:**

WB sensitivity for CL and VL was 100% and 91%, compared to IFA 4.6% and 87.8%, respectively. Sera from patients with CL and VL recognized numerous antigens with molecular weights ranging from 14 to 68 kDa and 12 to 94 kDa, respectively. The most sensitive antigens were 14 and 16 kDa for CL recognized by 100% of the sera from patients with proven CL and 12, 14 and 16 kDa for VL, recognized by 63.6%, 100% and 63.6% of the sera from patients with proven VL respectively. WB analysis is more sensitive than IFAT for the diagnosis of leishmaniasis particularly in cases of cutaneous leishmaniasis. The 12, 14 and 16 kDa can be valuable diagnostic molecules for serodiagnosis of leishmaniasis because at least two immunogenic molecules were simultaneously detected by all patient sera, as well as produced antibodies against these antigens have no cross-reactivity with other control groups.

**Conclusion:**

WB could be useful for screening and serodiagnosis of CL and VL in epidemiologic studies in endemic areas.

## Introduction

Leishmaniasis is a neglected tropical diseases caused by the *Leishmania* species, and covers a disease spectrum from a self-resolving cutaneous ulcer by *L. aethropica* and *L. tropica* complex in Old World and *L. mexi*c*ana* complex in the New World to a mutilating mucocutaneous due to *L. braziliensis* complex disease or a lethal visceral systemic illness due to species of the *L. donovani* complex (1–3). Approximately, 0.2 to 0.4 million of new visceral leishmaniasis (VL) cases and 0.7 to 1.2 million of new cutaneous leishmaniasis (CL) cases occur each year worldwide (4). The annual incidence of CL in Iran is estimated at approximately 20000 new cases, whereas this value for VL is much lower, approximately 100–300 new cases in endemic area (5).

The first-choice procedure for the diagnosis of CL is the microscopic demonstration of the *Leishmania* organism in the lesion aspirate, scraping, or biopsy specimen (6). However, the accuracy of microscopic examination is associated with various criteria including ability of the laboratory technician and the quality of the used reagents (7). Moreover, low and variable sensitivity and the need for invasive sampling techniques are major problems in such conventional methods. The routine methods for diagnosis of VL are splenic aspirates, in vitro culture of bone marrow and direct observation. However, these approaches are time-consuming, invasive and life threatening, unable to detect of the infected asymptomatic persons who may serve as a reservoir of VL, moreover in vitro parasite isolation is difficult and time-consuming (8).

The immunodiagnoses have become an important alternative for demonstrating the presence of parasite (9, 10). Several serological techniques based on immunologic response such as direct agglutination test (DAT), indirect immunofluorescence test (IFAT) and ELISA have been broadly developed in order to make a diagnosis of the leishmaniases (10–12). Some problem with serological tests for diagnosing are included; low specificity, false-positive results, and their low sensitivity in cases that the titers of antibodies against of leishmaniasis is low, such as HIV-positive patients (8, 9, 13).

Western blot (WB) technique is considered more sensitive and specific than IFA and ELISA, particularly in cases with low antibody titration, asymptomatic VL and CL (8, 14–16). In addition, WB provides detailed antibody responses to various leishmanial antigens and useful information about the parasite antigenic profile (17, 18). Moreover, it is useful when low serum antibody titers are present and has been proven to be highly specific for the diagnosis of different form of leishmaniases (15, 19).

Considering the above-mentioned points, the main goal of this study is to define *L. infantum* antigen that might be used in the diagnosis of VL and CL disease by WB technique and compare it with IFAT.

## Materials and Methods

### Ethical aspects

This study received approval from the local health authorities and the Shahid Beheshti University of Medical Science Ethical Committee. All patients were informed about the study and a written informed consent was obtained. Moreover, written and signed informed consents were taken from minors through their legal guardian or parents.

### Sampling

The serum samples used in this study were divided into four groups. These sera were collected from 2010 to 2014 in collaboration with Protozoology Unit of the School of Public Health, Tehran University of Medical Sciences (TUMS), Tehran, Iran.

*Group I.* Forty-three sera from patients with a clinical diagnosis of cutaneous leishmaniases confirmed by skin culture and microscopically were obtained from the Esfahan County, Center of Iran. The species of *Leishmania* in all of these sera were *L.* major and mean duration of infections was 30.2 ± 8.4 d.
*Group II.* Thirty-three serum samples were collected from patients with visceral leishmaniasis, mainly from the Meshkin shahr area, northwest of Iran. These sera were obtained from TUMS by Professor Mehdi Mohebali. These patients were positive as clinically, parasitologically (microscopically) and serologically (DAT) we used of four dogs sera proven for visceral leishmaniasis.
*Group III.* Thirty-nine sera were obtained from patients infected with intestinal parasites, toxoplasmosis and hydatid cyst. None of these patients had any lesions or antibodies suggestive of leishmaniasis.
*Group IV.* Twenty-three sera were obtained from healthy individuals from non-endemic areas for leishmaniasis. These individuals were examined with IFAT, DAT and formalin-ether concentration. No anti- *Leishmania* antibodies were detected in the control group. In addition, these individuals were detected negative for intestinal parasites, toxoplasmosis and hydatid cyst by Formalin-ether and serological methods.

### IFA antigen preparation and performance

Iranian strain of *L. infantum*; MCAN/IR/07/Moheb-gh, (GenBank accession No. FJ555210) were cultured in RPMI1640. Logarithmic phase of promastigotes was harvested after 72 h and a suspension at a concentration of 1 × 10^7^ cells/ml in phosphate buffered saline (PBS) (0.1 M phosphate, 0.33M NaCl, pH =7.2) was prepared. The promastigotes were washed and fixed with cold acetone on multi-well slides and kept stored at −20 °C until used. IFAT test was performed according to the method (20). Finally, slides were observed under immunofluorescent microscope (Zeiss, Germany) and the titers of 1:20 and above for CL and 1:80 and above for VL were considered positive.

### Western blot

Antigen preparation, SDS-polyacrylamide, gel electrophoresis and WB were applied (8) with slight modification. Briefly, promastigotes of *L. infantum* in logarithmic phase were harvested, washed twice with phosphate-buffered saline (PBS), and disrupted by sonication (Hielscher Ultrasonics UP200H, Germany). The sonicated homogenate was centrifuged at 1000 × g for 15 min at 4 °C after the supernatant collected and the protein concentration in the supernatants determined by method of Bradford (1976). Promastigote ly-sates were fractioned individually on a 15% SDS-PAGE gel. Antigens (4 mg/ml) were electrophoresed per gel width and subsequently transferred onto a nitrocellulose membrane (Whatman, Sigma Aldrich). Nitrocellulose strips were incubated with 1:100 diluted sera for 90 min, and then washed in PBS-0.2% Tween 20. The blots were incubated with 1:10000 diluted of anti-Human IgG–HRP conjugated (Bahar Lab, Iran) in blocking buffer for 90 min at room temperature. Enzymatic activity was revealed using Diaminobenzidine tetrahydrochloride (DAB) (Sigma-Aldrich) as chromogenic substrates.

### Repeating tests

After preliminary tests, the sera were stored at −20°C. After 6 months, we repeated IFA and WB tests on 29 of the sera of patients diagnosed as positive for VL by both tested in preliminary tests. In this study, DAT and skin culture considered as the gold standard for visceral and cutaneous leishmaniasis, respectively.

### Statistical analyses

Data analysis was carried out using the SPSS software ver. 21 (SPSS, Chicago, IL, USA). The frequency percentage was used to describe the positive and negative samples by two methods and for frequency of recognized antigens in each group of patients. Sensitivity for western blot and IFAT was defined as the number of samples with a western blot or IFAT-positive result/the number of samples with a positive diagnosis by DAT and skin culture × 100.

## Results

### Cutaneous leishmaniasis

All of the 43 sera from patients with CL were positive by WB analysis indicating a sensitivity of 100%, whereas only 2 samples were positive by IFAT giving a sensitivity of 4.6% (Table 1).

**Table 1:** Comparison of the IFAT and Western blot analysis of 43 serum samples from patients with proven cutaneous leishmaniasis by skin culture and microscopically

**Methods**	**No. (%) of sera**
**Western blot**	
Protein fraction 14 kDa	43 (100)
Protein fraction 16 kDa	43 (100)
Protein fraction 14 or 16 kDa	43 (100)
Protein fraction 14 and 16 kDa	43 (100)
**IFAT**	
Positive (cut-off >1/80)	2 (4.6)
Negative	41 (95.3)

In the sera of infected patients, many antigens were identified, which molecular weight had ranged from 14 to 68 kDa. The most frequently recognized bands 14–16 kDa were considered as diagnostic antigens for cutaneous leishmaniasis. In this group, 43 samples (100%) were reacted against the 14–16 kDa antigens. In addition, 41 (95%), 38 (88.3%) and 37 (86%) samples reacted against the 26–28 kDa, 42–44 kDa and the 36 kDa antigens, respectively. Other recognized antigens are shown in Table 2.

**Table 2:** Frequency of recognition of the major *Leishmania infantum* antigens by sera of 33 patients with visceral leishmaniasis, 43 patients with cutaneous leishmaniasis, 39 patients without leishmaniasis and 23 healthy individuals*

**% Recognition**				
**Antigen (kDa)**	**Patients with visceral leishmaniasis**	**Patients with cutaneous leishmaniasis**	**Patients without leishmaniasis**	**Healthy individuals**
92–94	21.2			
86			10.2	
70–72	12.1			8.6
66–68	39.3	76.7	15.3	8.6
56–58	30.3			
52–54		48.8		21.7
50–52				8.6
46–48				39.1
42–44	66.6	88.3	76.9	73.9
34–36	100	83.7	23	52.1
32	33	79	15.3	17.3
26–28		95.3	20.5	26
22–24	87.8	65	84.6	82.6
18–20		53	10.2	
16	63.6	100		
14	100	100		
12	63.6			

* Leishmanial antigenic bands of the12 kDa, 14-kDa and 16-kDa antigens in visceral leishmaniasis patients and 14-kD and 16-kDa in cutaneous leishmaniasis patients defined as diagnostic antigenic bands.

### Visceral leishmaniasis

**Fig. 1:**
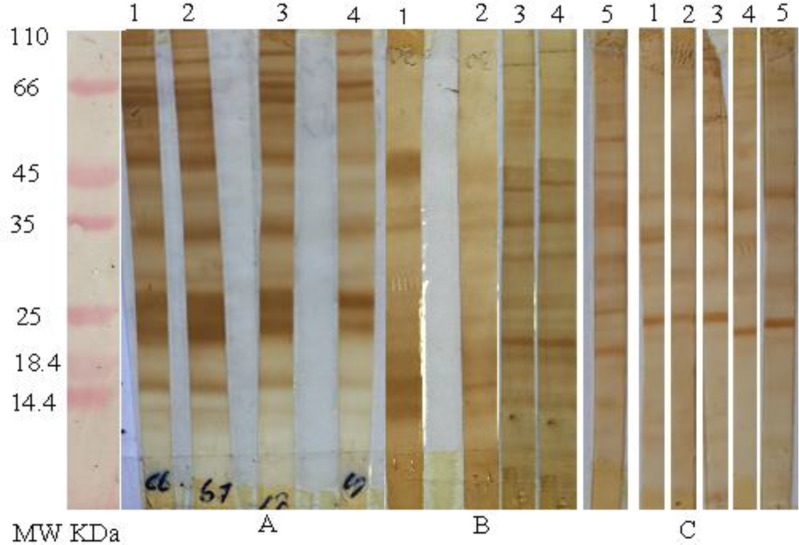
Western blot analysis of sodium dodecyl sulfate_polyacrylamide gel electrophoresis of promastigote forms of the *Leishmania infantum* strain recognized by **A,** serum samples from patients with visceral leishmaniasis, **B,** serum samples from patients with cutaneous leishmaniasis **C,** serum samples from patients without leishmaniasis.

Of 33 VL-infected sera, twenty-nine (87.8%) and 30 (90.9%) were positive by IFAT and WB analysis, respectively (Table 3). In addition, all of dog sera with VL were positive by both mentioned methods. The electrophoretic analysis of these sera demonstrates the presence of several bands with a molecular weight ranging from 12 to 94 kDa (Fig. 1).

**Table 3:** Comparison of the IFAT and Western blot analysis of 33 serum samples from patients with visceral leishmaniasis confirmed by direct agglutination test (DAT)

**Methods**	**No. (%) of sera**
**Western blot**	
Protein fraction 12 kDa	21 (63.6)
Protein fraction 14 kDa	30 (90.9)
Protein fraction 16 kDa	21 (63.6)
Protein fraction 12, 14 and 16 kDa	30 (90.9)
**IFAT**	
Positive (cut-off >1/80)	29 (87.8)
Negative	4 (12.2)

The most frequently recognized bands 12–14 and 16 kDa are considered as diagnostic antigens for VL. Among the 29 sera that were positive by both IFA and DAT, 29 (100%) sera reacted against the 14kDa and 20 (68%) sera reacted against 12 and 16 kDa antigens. At least two bands of the three 12, 14 and 16 kDa bands were observed in 100% of cases.

Results of the WB method on four human sera that negative for IFA showed similar bands only in one sample. Other recognized antigen presented in Table 2. WB showed that all of the sera from infected dogs with VL reacted with a several molecular weight antigens of *L. infantum* promastigotes (12–14, 16, 22, 32, 42, 50–52, 60–62, 66, 72–74 kDa).

### Control samples

WB analysis on 23 healthy subjects and 39 patients with other diseases showed several antigenic bands with a molecular weight ranging from 18 to 78 kDa. Of these samples, 75%–85% reacted with 22–24 and 42–44 kDa antigens. In addition, 20%–52% reacted with 34–36 kDa antigens. None of these sera showed diagnostic antigen bands of 12, 14 and 16 kDa. Results obtained in the WB with *L. infantum* antigens for different groups are presented in Table 4.

**Table 4:** Results obtained in the Western blot with *Leishmania infantum* antigens for different group studied*

			Western blot*			
			**P**		**N**	
			**NO.**	**%**	**NO.**	**%**
**Serum samples**	**Laboratory diagnosis**	**No. of serum samples**				
Group I^a^	cutaneous leishmaniasis	43	43	100	0	0.00
Group II^b^	visceral leishmaniasis	33	30	90.9	3	9.1
Group III^c^	Intestinal parasite	21	0	0.00	21	100
	Toxoplasmosis	13	0	0.00	13	100
	Hydatid cyst	6	0	0.00	5	100
Group IV^d^	Healthy individuals	23	0	0.00	23	100

* P _ positive when the sample recognized at least one antigenic band from a group of diagnostic antigen bands for CL and VL; N_ negative when the sample showed no reactivity.

a: Samples from patients with cutaneous leishmaniasis.

b: Samples from patients with visceral leishmaniasis.

c: Samples from patients with other parasitic diseases.

d: Samples from healthy individuals.

### Repeating tests

In repeating tests, we observed a decrease in the sensitivity of the IFAT after 6 months. Of the 29 tested sera, Twenty-six (89.6) were positive by IFAT, whereas all of the sera (100%) were positive by WB. Pattern of antigen bands was similar to preliminary tests.

## Discussion

Our results show that the sensitivities of WB analysis were 100% and 91% for the diagnosis of CL and VL, whereas these values for IFAT were 2.6% and 87.8% for CL and VL, respectively. These results are in agreement with previous studies, suggesting that WB is more sensitive than IFAT for diagnosis of CL and VL (15, 21).

Our results showed that seroreactivity with low-molecular-mass bands emerged (12–14 and 16 kDa) as a valuable marker for diagnosis if CL and VL. Similar to our findings in this study, these bands were recognized as specific antigens in previous studies (8, 15, 22, 23)

Antigen bands ranging 14–16 kDa were most recognized antigens in HIV-*Leishmania-*coinfected humans (8). Similar results were observed in our study which antigens of 14 and 16 kDa were recognized in 100% and 63.6% of the patients’ sera, respectively. In our study, these antigenic bands were not detected in control groups; cross-reactivity was 2% (8). Moreover, in the present study antigen of 12 kDa was recognized in 63.6% of patients’ sera and not detected in control groups, thus we considered antigen of 12 kDa as diagnostic antigen for VL.

In addition to human, other studies in naturally and experimentally infected dogs have described that 12–16 kDa immunodominant bands appeared as most specific antigens recognized by WB analysis (22, 23).

In present study, other antigens frequently recognized in VL group were those with molecular weights of 22–24, 34–36, 42–44 and 66–68 kDa. T antigens were also recognized in control groups indicating they are non-specific antigens. In current study, WB showed specificity of 100%, although sera of control groups were from the non-endemic area. Similar results were observed in serum samples from dog with visceral leishmaniasis (22). A slight decrease occurred in specificity of WB in our study. An explanation could be sampled collection from endemic areas. A similar result was obtained in which specificity of 98% was reported for western blotting in serodiagnosis of VL (8). We repeated IFAT and WB for some of patient’s sera with VL that were positive by both WB and IFAT tested at 6 months after preliminary experiments. Taking into account the 29 sera re-examined, results showed a reduced sensitivity for IFAT (26/29, 89.6%), whereas we did not observe any change in the pattern of WB results (29/29, 100%). This result can be indicating more sensitivity of WB than IFAT. WB is capable incorrect diagnosis of VL even at an antibody concentration of 10^−9^ μg/ml, whereas the ELISA had a detection limit of 10^−1^ μg/ml and the IFAT had a detection limit of 10^−2^ μg/ml (8).

In cases of CL, our study showed a sensitivity and specificity of 100% for WB as 14 or 16 kDa Ag considered as antigenic bands. The *L. infantum* Ag was tested for the detection of CL caused by *L. major* and found bands of 14 and 18 kDa as antigenic bands in patients’ sera. They reported sensitivity of 100% and 47% for WB and IFAT, respectively. In their study, specificity for WB was 96% whereas this value in our study was 100% (sampling of control group carried out in non-endemic area in both studies) (15). Bands between 15 kDa and 118 kDa were detected when they used *L. infantum* as the antigen sources for the diagnosis of CL caused by *L. tropica.* They reported the sensitivity of 99.1% and 100% for WB and 78% and 93% for ELISA in diagnosis of CL and VL, respectively (24). WB using was examined different *Leishmania* species as the antigen sources for the diagnosis of American tegumentary leishmaniasis (ATL). They reported antigen bands between 13 kDa and 150 kDa. No specific Ag was recognized by 100% of the serum samples from ATL patients. In addition, they showed that a mixed human infection of *T. cruzi* and *Leishmania* species could be detected by WB (25). The mucosal leishmaniasis sera generally reacted intensely to antigens of 75, 66, and 45 kDa and weakly to 48–50 kDa antigens, whereas cutaneous leishmaniasis sera generally reacted weakly to antigens of 45, 66, and 75 kDa and intensely to 48- to 50-kDa antigens (26). In our study, antigen bands of 18–20, 22–24, 26–28, 34–36, 42–44 and 66–68 kDa were observed highly in patients with CL, however, these antigens observed also in control groups. We considered these fractions as non-specific. Similar to our results, the presence of antibodies against 14 and 18 kDa antigens are important for the diagnosis of symptomatic and asymptomatic infections, they introduced that WB is an effective method for the detection of asymptomatic patients in epidemiological areas (16). Slight alterations in the WB technique and different source of antigen could result to the different migrations of diagnostic bands in different studies.

There were some limitations to our study such as low sample size of positive patients and lack determines the stage of disease because the limitation to access the patients. However, due to the lack of enough financial funding, we could not use of other serological and molecular techniques such as ELISA, Montenegro test and PCR methods for more accurate to determine of sensitivity and specificity.

## Conclusion

WB analysis is more sensitive than IFAT for diagnosis of leishmaniasis particularly in cases of cutaneous leishmaniasis. The 14 and 16 kDa antigens for CL and 12, 14 and 16 kDa antigens for VL showed that can be valuable diagnostic molecules for serodiagnosis of leishmaniasis, because at least one or several immunogenic molecules were simultaneously detected by all patient sera, as well as produced antibodies against these antigens have no cross-reactivity with other control groups. Further studies are required to evaluate the usefulness of WB analysis in diagnosis CL and VL in epidemiological study.
